# A multicenter retrospective study on severe complications of acute otitis media and sinusitis in healthy children in the context of covid pandemic – CAOS-kids

**DOI:** 10.1038/s41598-026-67005-y

**Published:** 2026-08-21

**Authors:** Kornelia E. Andorfer, Torstein Grønseth, Juned Zafar Butt, Randall A. Bly, Jeremy S. Ruthberg, Michael Habenbacher, Peter Valentin Tomazic, Fatemeh Kashani, Maria Teresa Arcuri, Bresciani Lorenzo, Marie Gisselsson-Solén, Isabella Sjölander, Sara Zenk, Valentinos Sofokleous, Ivan Delchev, Florian Zeman, Christopher Bohr, Kathrin Renner

**Affiliations:** 1 Department of Otorhinolaryngology, University Hospital Regensburg, Franz-Josef-Strauss-Allee 11, 93053 Regensburg, Germany; 2 Department of Otolaryngology, Head and Neck Surgery, Oslo University Hospital, Oslo, Norway; 3 Department of Otolaryngology, University of Washington, Seattle Children’s Hospital, Seattle, WA USA; 4 Department of Otolaryngology-Head and Neck Surgery, University of Washington, Seattle, WA USA; 5 Department of Otorhinolaryngology, Medical University of Graz, Graz, Austria; 6 Department of Otorhinolaryngology, Head and Neck Surgery, University Hospital LMU, Munich, Germany; 7 Unit of Otorhinolaryngology-Head and Neck Surgery, Department of Surgical Specialties, Radiological Sciences, and Public Health, University of Brescia, Brescia, Italy; 8 Department of Otorhinolaryngology, Head and Neck Surgery, Lund University, Lund, Sweden; 9 Department of ENT, Surgical Science, Uppsala University Hospital, Uppsala, Sweden; 10 Department of Pediatric Otorhinolaryngology, Athens Childrens Hospital P. A. Kyriakou, Athens, Greece; 11 Medical University of Plovdiv, Plovdiv, Bulgaria; 12 Center for Clinical studies, University Hospital Regensburg, Regensburg, Germany

**Keywords:** Covid-19, Pediatrics, Immune system, Brain abscess, Otitis, Sinusitis, Diseases, Health care, Medical research, Microbiology

## Abstract

Intracranial sinogenic and otogenic complications and high-grade orbital complications of sinusitis in children are rare, but case numbers appeared to increase after COVID-19 health measures were lifted. As similar trends were reported internationally, we conducted a multicenter retrospective case series to evaluate this “perceived accumulation”. The study population included otherwise healthy children treated at eight participating centers from seven countries including a period from 2012 to 2023 for above-mentioned complications. Recorded parameters included age, diagnosis, timing, duration and type of inpatient therapy, as well as microbiological and virological findings, and differential blood count. A total of 289 data sets were received from the eight centers. Cases per semiannual increased significantly from 6 to 28 in the sinogenic and 3 to 12 in the otogenic cohort comparing pre- to post-pandemic period. Main patient characteristics were preserved. However, the number of surgical interventions per patient rose slightly in the post-pandemic sinogenic cohort. No association with acute COVID-19 infection was identified. In summary, intracranial sinogenic and otogenic complications in children rose significantly after the lifting of COVID-19-related restrictions. As immunity to respiratory viruses is not permanent, COVID-19 protective measures may have resulted in a lack of stimulation of the immune system, favoring the occurrence of superinfections. This highlights the importance of global respiratory infection research and surveillance programs, as well as open data sharing and maintaining vaccine development and use.

**Supplementary Information:**

The online version contains supplementary material available at 10.1038/s41598-026-67005-y.

## Introduction

According to literature about 5–10% of upper respiratory tract infections in pediatric patients are complicated by acute bacterial sinusitis^1,2^. Most cases of bacterial sinusitis resolve with antibiotic therapy^3^. However, acute Rhinosinusitis can be complicated by periorbital, intracranial and, more rarely, osseous involvement. According to the European Position Paper on Rhinosinusitis and Nasal Polyps 2020, the incidence of complications from acute bacterial sinusitis is 3/1,000,000 per year^4^. It is estimated that approximately 3% of pediatric patients hospitalized for acute sinusitis present with an intracranial complication^5^. Regarding otogenic complications (e.g. intracranial, vascular, osseous) incidences range from 0.13% to 1.97%^6,7^.

At the University Hospital Regensburg, children and adolescents with intracranial sinogenic and otogenic complications compose a very rare entity. In the second half-year of 2022, however, this rapidly changed. A rising number of children and adolescents had to be medically treated due to severe complications with sinogenic foci. This observation was in line with the development of intracranial complications of otogenic origin. Interestingly, colleagues from several different nations also reported an accumulation of the aforementioned complications. For example, at an US children`s hospital, there was more than a two-fold increase in sinogenic and otogenic complications observed among children^8^. Thus, a data retrieval was launched by the Emergency Infections Network with data from 8 institutions suggesting a possible increase in some forms of intracranial infections in persons aged ≤ 18 years living in the United States during March 2020–March 2022. In view of this development, Issa et al. (2024) performed a small retrospective single-center study revealing a 5-fold increase in pediatric otogenic and sinogenic intracranial abscesses in the context of the COVID-19 pandemic^9^. Several single-center studies conducted both within and outside Europe have corroborated this trend^10,11^.

These publications, together with the observation at our own clinic, were impetus to initiate a retrospective multicenter cohort study based on a chart review to further quantify this perceived accumulation in a larger collective from several different countries. We hypothesized that there is a higher frequency of intracranial complications of sinusitis and otitis (ICSO) and high-grade orbital complications of sinusitis after cessation of the COVID-19 protective measures.

We further aimed to assess whether the spectrum and severity of complications, the duration of hospitalization, and the underlying microbial profile had changed, and whether these changes might be directly or indirectly associated with SARS-CoV-2 infection.

## Materials and methods

### Study design and setting

The study was designed as a retrospective multicenter case series based on chart review. Eight centers across Austria, Germany, Greece, Italy, Norway, Sweden, and the USA participated and delivered full data sets. These datasets were pooled and analyzed jointly. In addition, a restricted dataset from a second Swedish center was available, providing case numbers, patient age and sex, and type of diagnosis for the period from 2017 to 2023. We aimed to adhere to the STROBE guidelines in reporting. This study was conducted in accordance with the Declaration of Helsinki and approved by the Ethics Committee of the University of Regensburg (Institutional Review Board; IRB; approval number: 23–3442-104). The data did not contain any personal identifiers, so that no conclusions could be drawn to the individual patients surveyed.

### Ethical approval and data collection

Anonymized datasets were retrospectively collected at eight centers using standardized data acquisition forms being distributed to each participating center. Data were obtained exclusively from routine clinical care as documented in the individual patients’ medical records, and no additional interventions were performed for study purposes. In line with Sect. 27 (4) of the Bavarian Hospital Act, the Institutional Review Board (Ethics Committee of the University of Regensburg) waived the requirement for written informed consent because only anonymized retrospective data were analyzed. The Ethics Committee stated that there were no ethical or legal objections to the conduct of this research project.

### Variables and data completeness

From the eight centers that provided complete datasets, the following variables were recorded: age, sex, type of diagnosis, time point of inpatient admission (categorized by half-year), length of hospital stay, and type of inpatient therapy. Complications and the occurrence of acute COVID-19 infection was also recorded. These data sets were complete. Where available, data on differential blood count at the time of acute infection were extracted (158/190 patients in the sinogenic cohort and 80/99 patients in the otogenic cohort). Microbiological and virological findings, as well as the presence of acute and a history of previous COVID-19 infection(s), were requested from all centers. Information on bacterial infection was available for 149/190 (approx. 78%) of patients in the sinogenic cohort and 81/99 (approx. 82%) of patients in the otogenic cohort, whereas data on viral infections were sparse (32/190 patients in the sinogenic cohort) and therefore incomplete.

### Inclusion and exclusion criteria

Patients were included irrespective of sex, provided that sufficient clinical information was available to confirm the diagnosis and assign the case to the appropriate observation period. The study population comprised children and adolescents aged 0–17 years, who were treated at the participating centers between 1 January 2012 and 31 December 2023. Eligible patients had intracranial or orbital (at least Chandler 3) complications of sinogenic origin (*N* = 190 with complete datasets; *N* = 224 including an additional Swedish center providing a restricted dataset) or otogenic origin (*N* = 99). Diagnoses were established based on physical examination and radiologic findings. The following complications were included: Mastoiditis, Potts Puffy tumor, meningitis, sinus vein thrombosis (not including cavernous sinus thrombosis), epidural abscess, subdural abscess, brain abscess, encephalitis and orbital complication according to the Chandler classification (Chandler 3 = subperiosteal abscess/Chandler 4 = orbital abscess/Chandler 5 = cavernous sinus thrombosis).

Cases were identified retrospectively through hospital information systems and medical records at each center. Patients were excluded if key clinical information was missing (example e.g. incomplete documentation of the diagnosis or of the timing of inpatient admission). In addition, patients were excluded if their medical history at the time of data collection indicated any of the following: : current or previous diagnosis of cancer, immunosuppression, autoimmune disease, adiposity, chronic illnesses, a history of fractures involving the midface, skull base or temporal bone, meningocele or encephalocele, and craniofacial dysmorphia.

The final study population comprised all eligible cases from the datasets of the eight main centers plus the aggregated cases from the restricted Swedish dataset.

### Detection of bacterial and viral infections

Analysis of microbial infection, determined either in blood or smear, was included. Analysis of bacterial infection was conducted routinely, while virus determination was performed on an occasional basis. Microbial infection was identified using a standard institutional protocol, including rapid antigen swab and respiratory viral panel; COVID-19 testing was performed via rapid swab or multiplex respiratory viral PCR, depending on the center.

### Differential blood count

The distribution of the leukocyte populations in the peripheral blood was determined in a routine differential blood count.

### Statistical analysis

The observation period was divided into three phases: a pre-pandemic period (from the first half of 2012 until December 2019), a pandemic period (from the first half of 2020 until the first half of 2022), and a post-pandemic period (from the second half of 2022 until the end of 2023). Case numbers per semiannual (also referred to as case frequency or cases per semiannual) were analyzed on pooled analyses across all centers and in addition on a per-country basis for the sinogenic cohort. Despite the analysis of case frequencies, we focused on the statistical comparison of pre- to post-pandemic subgroups, due to the inconsistent COVID-19 protective measures in the different countries. Statistical significances were calculated applying the Mann-Whitney test for two group comparisons, Fischer’s exact tests for categorical data using GraphPad Prism 10.4. The average case count per half-year within each time interval (pre-pandemic, pandemic, post-pandemic) was calculated by dividing the number of cases by the number of half-years in each interval. Comparisons between time intervals were performed using rate ratios and the exact Poisson test. Estimates are reported with their corresponding 95% confidence intervals.

## Results

### Number of cases

A retrospective study was conducted with eight centers from seven different countries in Europe and the USA participating, covering the period from 1st January 2012 through 31st December 2023. A total of 289 data sets were received (103 female, 185 male, 1 diverse). Of these, 190 (73 female, 117 male) were attributed to sinogenic and 99 (30 female, 68 male, 1 diverse) to otogenic complications. Patient characteristics are summarized in Table 1. Additionally, we received a restricted data set of another 34 patients (14 female, 20 male) from a second Swedish center (Supplementary Table 1), which were included only in analysis on case numbers. Patient groups displaying sinogenic and otogenic complications are referred to as sinogenic and otogenic group/cohort.

**Table 1:** Patient characteristics of the sinogenic and otogenic cohorts from the eight centers delivering full data sets. For age the median and interquartile range (IQR), for hospitalisation, the median and SD are displayed. Statistical significances were calculated applying the Mann Whitney test for two group comparisons (age, hospitalization), and for categorical data the Fisher’s exact test (gender).

Sinogenic cohort					
Demographic data	Total	Pre-pandemic	Pandemic	Post-pandemic	p-value pre vs. post
Number of cases	190	86 (45%)	32 (17%)	72 (38%)	
Gender					0.51
Female	73 (38%)	31 (36%)	12 (37%)	30 (42%)	
Male	117 (62%)	55 (64%)	20 (63%)	42 (58%)	
Age (IQR)		7.5 (3.75–11.0)	9.5 (6.0–13.8)	9.5 (5–12)	0.06
Hospitalisation (days)		6 ± 13.2	11 ± 11.9	9 ± 8.9	0.19

Otogenic cohort					
Demographic data	Total	Pre-pandemic	Pandemic	Post-pandemic	p-value pre vs. post
Number of cases	99	47 (47%)	14 (14%)	38 (38%)	
Gender					0.64
Female	30 (30%)	16 (34%)	3 (21%)	11 (29%)	
Male	68 (69%)	30 (64%)	11 (79%)	27 (71%)	
Diverse	1 (1%)	1 (2%)	0 (0%)	0 (0%)	
Age (IQR)		4 (2.0–8.0)	3.5 (2.0–5.2)	4 (2.0–6.0)	0.29
Hospitalisation (days)		11 ± 16.3	12.5 ± 5.2	10 ± 7.7	0.77

**Fig. 1:**
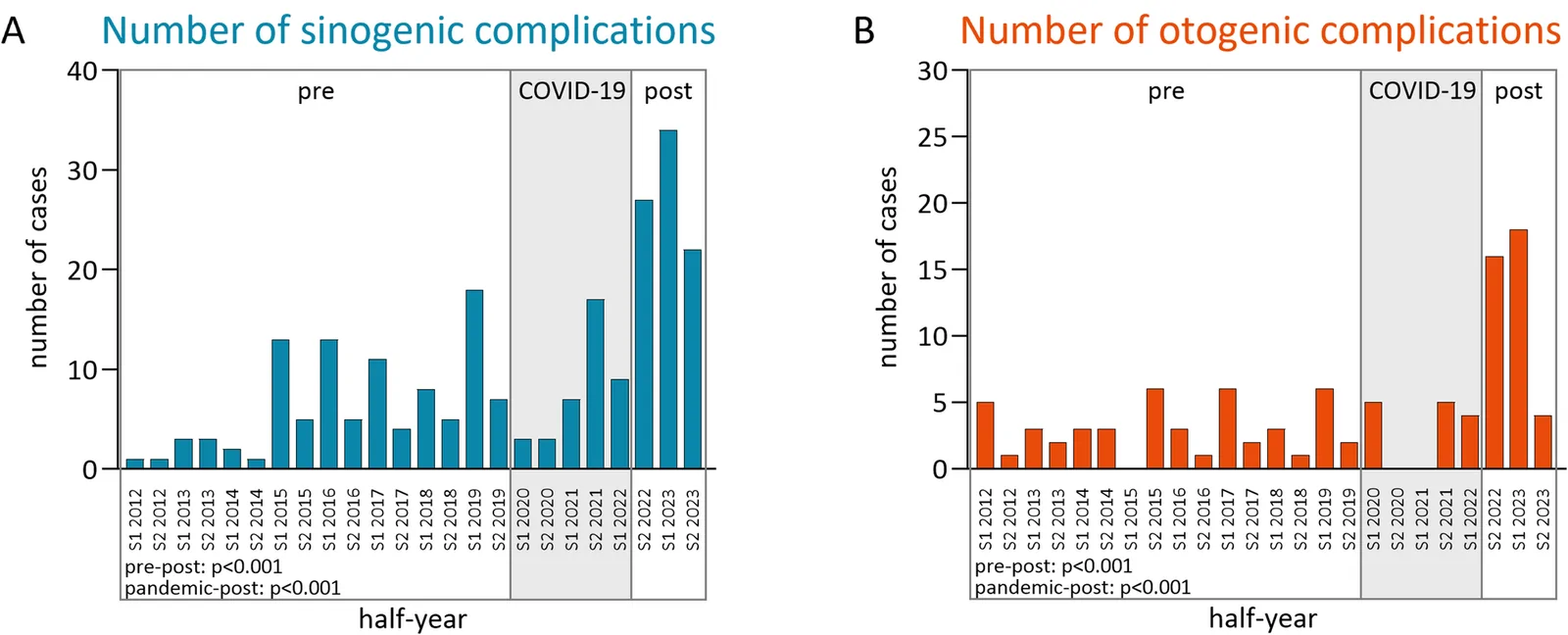
Increased number of patients per semiannual with intracranial and orbital complications in the post-pandemic period. From the beginning of 2012 to the end of 2023, data regarding the number of patients per semiannual with intracranial (**a**) sinogenic or (**b**) otogenic complications was collected from eight different centers. Displayed are cumulative counts. The average frequency within each time interval (pre-pandemic, pandemic, post-pandemic) was calculated by dividing the number of cases by the number of semiannuals in each interval. Comparisons between time intervals were performed using the exact Poisson test.

Prior to the onset of the Covid-19 pandemic, the mean case frequency of patients diagnosed with severe intracranial complications was approximately 6 per semiannual for the sinogenic group and 3 per semiannual for the otogenic group (Table 2). During the pandemic period, the case frequency of both, the sinogenic and otogenic group, remained relatively stable displaying rate ratios pre-pandemic vs. pandemic of 0.80 (95%-CI: 0.55, 1.19; *p* = 0.233) and 0.98 (95%-CI: 0.54, 1.89; *p* = 1.00), respectively. In the post-pandemic period, a marked and statistically significant surge was observed in both cohorts (Fig. 1A and B), reaching 28 cases per semiannual in the sinogenic group and 12 cases per semiannual in the otogenic group (Table 2). The significant increase was observed comparing the pre-pandemic to the post-pandemic (sinogenic cohort rate ratio = 0.22; 95%-CI: 0.16, 0.30; *p* < 0.001); otogenic cohort: 0.24 (95%-CI: 0.15, 0.38; *p* < 0.001) and the pandemic to the post-pandemic period (sinogenic cohort: rate ratio = 0.28; 95%-CI: 0.18, 0.41; *p* < 0.001); otogenic cohort: 0.24; 95%-CI: 0.12, 0.45; *p* < 0.001). A re-analysis of the sinogenic cohort was conducted, utilizing data from 2017 to 2019 as the pre-pandemic period, since one center from Sweden provided a data set that began in 2017. Statistical significances were comparable. In general, the prevalence was higher in the first half of the year in the pre-pandemic period. However, this phenomenon was not observed in the post-pandemic period. As corona restrictions varied between countries, this led us to analyze data on a per country base in the sinogenic cohort. However, an increase in case frequencies could also be observed in countries with less restrictive measures (Supplementary Table 2). Furthermore, no correlation could be established between the number of weeks that schools were closed in the participating countries and the intensity of increase in the respective case numbers.

**Table 2:** Mean frequencies in the sinogenic and otogenic cohort. Mean frequencies were calculated by dividing the number of cases in each period by the number of half-years.

Sinogenic cohort				
Period	#Half-years	#Cases	Frequency	95%-CI
Pre-pandemic	16	100	6.25	5.09–7.60
Pandemic	5	39	7.80	5.55–10.66
Post-pandemic	3	85	28.33	22.63–35.03

Otogenic cohort				
Period	#Half-years	#Cases	Frequency	95%-CI
Pre-pandemic	16	47	2.94	2.16–3.91
Pandemic	5	15	3.00	1.68–4.95
Post-pandemic	3	37	12.33	8.68–17.00

The subsequent objective was to investigate whether the increase in cases per semiannual was accompanied by a change in patient characteristics.

### Age and gender

Intracranial complications of sinogenic origin are prevalent among older children and young adults due to the delayed development of the frontal sinus^12^. According, the median age of the children in the sinogenic cohort was 8.5 years. Notably, we detected an increase in age, rising from 7.5 years before the onset of the COVID-19 pandemic to 9.5 years during and after pandemic (Table 1). Male predominance has been repeatedly described in literature and is explained with a more complex sinonasal anatomy^13–15^. In addition, studies suggest that girls’ immune systems develop earlier than boys’, resulting in women developing stronger immune responses and antibodies to fight infectious pathogens^16^. Accordingly, the majority of these patients was male in all periods analyzed (Table 1). In the otogenic group the median age of approximately 4 years remained stable. Furthermore, we recorded again a male predominance of 69%. Taken together, in terms of age and gender distribution, no significant differences were detected among periods investigated (Table 1).

### Assessment of disease severity

In order to assess the severity of the disease, length of hospitalisation, number and type of surgical measures and complications were evaluated. In the sinogenic group the length of inpatient stay (hospitalization) increased over time with a median of 6 days before and 9 days after the pandemic (Table 1). However, this increase was already evident during the pandemic. The otogenic group demonstrated stability (Table 1). We observed an increase in surgical interventions in the sinogenic group. This observation translated into a slight but significant increase in interventions per patient (0.9 to 1.2 per patient, *p* = 0.03). No differences in type and frequency of surgical measures were detected between the pre- and post-pandemic otogenic subgroups (Table 3).

**Table 3:** Therapeutic interventions in the sinogenic and otogenic cohorts.

Sinogenic subgroup						
	Treatment					
Period	Antibiotics	FESS	Open sinus surgery	Bure hole	Craniotomy	Total
Pre-pandemic (%)	85 (100)	56 (65)	8 (9)	2 (2)	15 (17)	81
Post-pandemic (%)	72 (100)	57 (79)	11 (15)	3 (4)	14 (19)	85

Otogenic subgroup						
	Treatment					
Period	Antibiotics	Myringotomy	Mastoid-ectomy	Burrhole trepanation	Craniotomy	Total
Pre-pandemic (%)	47 (100)	38 (81)	37 (79)	0 (0)	5 (11)	80
Post-pandemic (%)	38 (100)	34 (90)	26 (68)	1 (3)	2 (5)	63

**Fig. 2:**
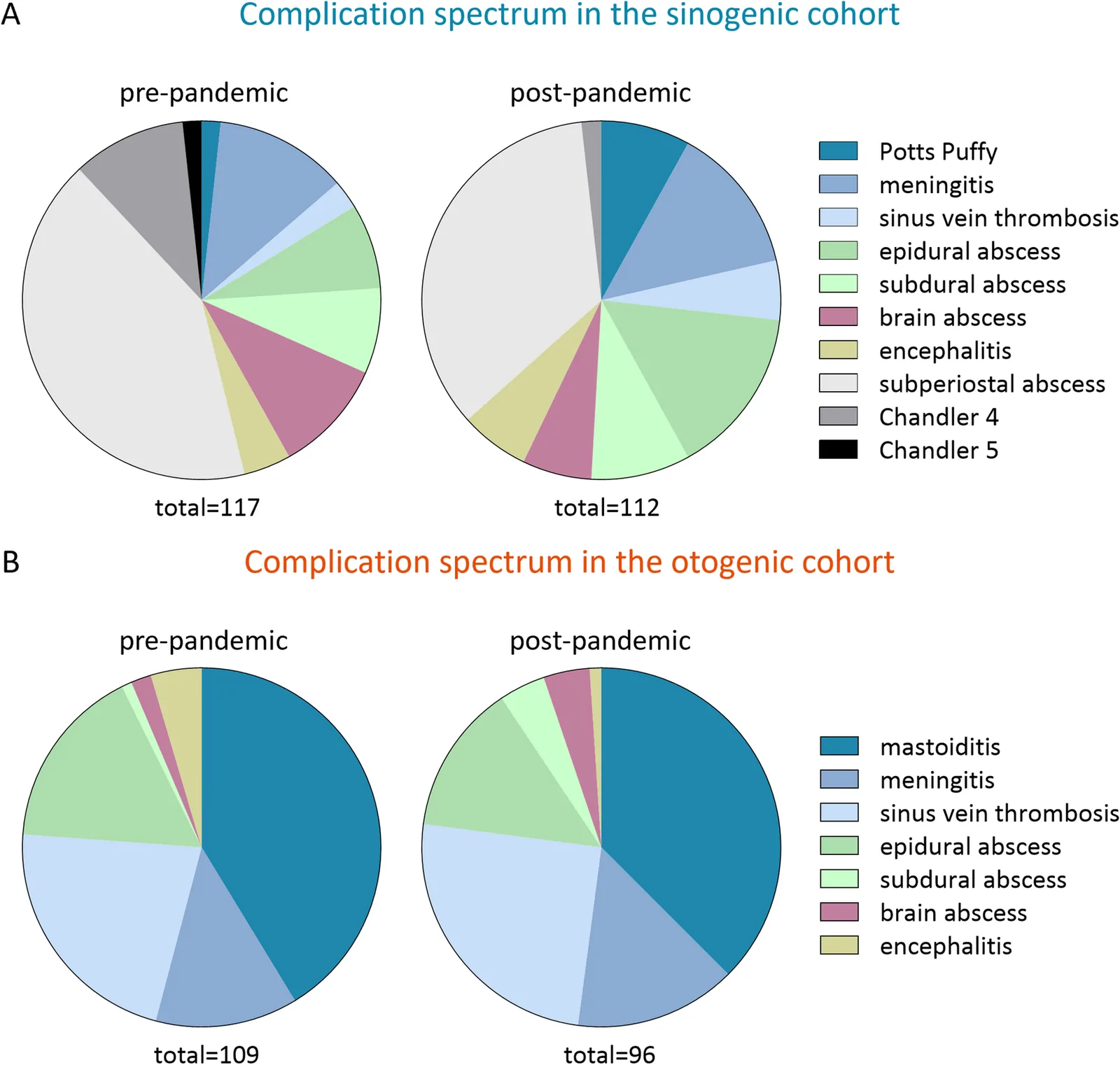
Type and frequency of complications in the pre- and post-pandemic subgroups. Data regarding complications were received for all patients included in the study. Shown are type and frequency of complications recorded in the (**a**) sinogenic and (**b**) otogenic pre- and post-pandemic subgroups. Statistical significance was tested by Fisher exact test including the four most frequent bacterial species.

Next, we analyzed whether the number and type of complications had changed. The data refer to concurrent complications during a single hospitalization in each case. A total of 280 complications in the sinogenic and 235 in the otogenic group were documented. The type and frequency of these complications are summarized in Fig. 2A and B. The number of complications per patient was not different between the pre- and post-pandemic subgroups (sinogenic 1.33 vs. 1.55, *p* = 0.09; otogenic 4.0 vs. 4.2, *p* = 0.19). Within the sinogenic cohort, the proportion of the 5 most frequent complications remained consistent, with subperiosteal abscess (Chandler 3) (pre: 42% vs. post: 35%) and meningitis (pre: 12% vs. post: 13%) being the most prevalent. Meningitis was solely recorded as an additional diagnosis. The most prevalent shifts were observed in the proportion of epidural abscesses (8% vs. 15%), the decrease in Chandler 4 (10% vs. 2%) and the increase in the prevalence of Pott’s puffy tumors (pre: 2% vs. post: 8%). In the otogenic cohort, the most frequently presented complications were mastoiditis (41% vs. 38%), cerebral sinus vein thrombosis (CSVT, pre: 22% vs. post: 25%), meningitis (pre: 13% vs. post: 15%), and epidural abscess (pre: 17% vs. post: 14%). Type and proportion of complications were comparable in the pre- and post-pandemic period. Finally, we examined the proportions of the major peripheral blood mononuclear cell populations. The infection-related shift towards neutrophils at the expense of the lymphocyte population was observed in both subgroups (Supplementary Fig. 1). In conclusion, patient characteristics in terms of age, gender and disease severity remained stable in the otogenic cohort and only slight changes were observed in the sinogenic cohort.

### Microbial spectrum

Sites tested smear or blood for the presence of bacterial strains in approximately 80% of patients. Although all patients received antibiotics (Table 2) a bacterial infection was still detectable in the majority (Table 1). From all patients evaluated, only one patient was detected negative in the sinogenic and 5 patients in the otogenic cohort. The bacterial spectrum detected exhibited a high degree of similarity between the sinogenic and the otogenic groups in terms of the main strains and species involved. Streptococci and staphylococci were the most prevalent. No changes were observed between the pre- and post-pandemic subgroups (Supplementary Fig. 2 A and 2B). Ongoing viral infections were only determined in a limited number of patients (31/190) in the sinogenic cohort. Among those, the most frequently detected virus was Influenza (56%) followed by Respiratory Syncytial Virus (RSV, 16%). The paucity of cases precludes any inferences regarding potential alterations in the viral spectrum and the possibility of a prior infection that has since abated cannot be ruled out. Nevertheless, from the start of the pandemic, patients were tested for the presence of a SARS-Cov-2 infection. Notably, the virus was detected in less than 5% of patients in the post-pandemic subgroups. Furthermore, a temporal correlation with a previous infection with SARS-Cov-2 could not be established, as only 4 patients reported a proven preceding infection, with the infection having occurred at least 4 months previously. According to literature, SARS-Cov-2 infection might have a correlation with the development of CSVT^17^. In our cohort the portion of CSVT among all complications was found to be comparable across the cohort (Fig. 2, sinogenic: pre: 3.7%, post: 8.3%, otogenic: pre: 51%, post: 63%).

In summary, we observed a marked increase in the cases per semiannual of pediatric patients presenting with sinogenic or otogenic complications in the post-pandemic period, whereas patient characteristics, the spectrum of complications, and the bacterial strains detected remained largely unchanged.

## Discussion

Beginning in the first half of 2020 until the first half-year of 2022, the COVID-19 pandemic was a major challenge, especially for children and adolescents. In an attempt to slow viral transmission, schools were temporarily closed, and social distancing and wearing masks became compulsory.

Moreover, the so-called NPIs (non-pharmacological interventions), had major consequences on the spread of other respiratory germs leading to viral or post-viral infections^18–20^.

As early as 2021, scientists predicted that the lack of stimulation of the immune system in the course of NPIs during COVID-19 pandemic will lead to an increase in infections once they are lifted^21^. This prognosis was later confirmed by numerous publications^22–24^. According to Feinmann et al. (2024) citing the UK based disease forecasting firm Airfinity, 44 countries have experienced a 10-fold increase in the incidence of at least one of 13 infectious diseases compared with a pre-pandemic baseline^25^.

On behalf of the European Society of Pediatric Neurosurgery (ESPN), Massimi et al. (2024) published a multicenter survey including 30 neurosurgical European centers. In their collective of 254 children, they reported a significant difference between the pre- and post-COVID period (28,3 cases/year versus 86 cases/year)^26^. Lately an even larger multicenter study on 638 children, being treated across 31 Neurosurgical centers in North America also reflected this trend with a maximum number of cases in the fourth quarter of 2022^27^.

The results of our retrospective multicenter analysis, including 289 generally healthy children in Europe and the USA, strongly supports the thesis of a growing post-pandemic susceptibility for bacterial infections and respectively superinfections. We observed a significant increase in the number of patients presenting with intracranial complications of otogenic (from 3 to 12 cases per semiannual) and sinogenic (from 6 to 28 cases per semiannual) in the post-pandemic period compared with the pre-pandemic period. Main patient characteristics, including age, sex, duration of hospital stay, and type of inpatient therapy, remained stable across the examined periods.

Several factors could have contributed to this trend. Immunity to certain viruses is not permanent and must first be established, especially in children and adolescents. NPI measures have led a lack of immune stimulation. Furthermore, the proportion of the „naive“ population to community-acquired pathogens resulted in a decline of herd immunity favoring the occurrence of viral infection and bacterial superinfections^21,28^. Physiological colonization with bacteria-like serotypes of Streptococcus pneumoniae happening in the first years of life helps the development of mucosal and systemic immunity^29^. With decreased circulation of those strains due to NPIs this protective effect is consequently lost. Cohen et al. have established the concept of „immunity debt“ for re-emerging of infectious diseases following the lifting of barrier measures. This concept is highly controversial and has been misinterpreted in both academic and popular science by equating a lack of immune stimulation with an immune deficiency^30^. A lack of immune stimulation due to a lack of exposure to germs means less “training” for the innate immune system. This limits its effectiveness and responsiveness^31^. Interestingly, even in Sweden, where NPIs were less restrictive, the increasing case frequencies of the observed complications appear to suggest a transient reduction in immune stimulation. This phenomenon may particularly affect adaptive immunity, which depends on repeated antigen exposure for the maintenance of immunological memory. In addition to the increased number of bacterial superinfections observed in our cohort, a delayed or less effective immune response resulting from insufficient immunological priming may have contributed to the greater severity of bacterial superinfections, as reflected by the higher proportion of complications and the increased need for surgical intervention, particularly in the sinogenic subgroup. Along with these indirect consequences of the COVID-19 pandemic, the question remains whether SARS-CoV-2 had a direct impact on the development of intracranial complications. Like other respiratory viruses, COVID-19 infections have been found to be responsible for the susceptibility to bacterial co-infections and secondary infections to a variable extent ranging from 2,5–41,9%^32–34^. In the present study the majority of patients were tested for the presence of a COVID-19 infection during the pandemic period. Notably, the post-pandemic test rate regarding COVID-19 was undefined. Consequently, it is not possible to draw any conclusions on the causative nature to sinogenic and otogenic complications. Nevertheless, even during the pandemic period only a minority of patients exhibited an acute infection. None of the patients had a positive test result for the infection in the weeks prior to the manifestation of intracranial complications. Given the potential association with SARS-CoV-2, the occurrence of cerebral sinus vein thrombosis (CSVT) merits particular consideration^35^. The mechanisms through which SARS-CoV-2 might trigger CSVT are not yet fully elucidated. Besides prothrombotic effects of unopposed angiotensin II, systemic inflammation, cytokine release and endothelial cell injury might support the hypercoagulable state in COVID-19^36^. In our cohorts, the proportion of patients experiencing CSVT only slightly increased in both subgroups. Given the limited number of patients with documented acute or past infection, a direct correlation with COVID-19 is unlikely. Surplus, patients being hospitalized because of acute COVID-19 infections rarely (about 7%) have bacterial co-infections^37^. Nevertheless, SARS-CoV-2 infection has been associated with sustained alterations in the immune system in other contexts. Changes within the innate immune system may persist for several months, extending up to one year following severe COVID-19 illness^38^. In addition, evidence indicates that approximately 10 months post-infection, patients may exhibit reduced absolute counts of neutrophils, monocytes, and lymphocytes^39^. This survey also depicts, that germs against which vaccinations are available, have played a viable role in post-pandemic re-emerging infections. It is worth mentioning that a high proportion of Streptococcus pneumoniae (pre 22.2%, post 38.7%) was detected in the otogenic subgroup. Hemophilus influenzae, RSV and Influenza were detected as well. During the early pandemic, scientists strongly recommended rapid catchup vaccination programs to protect children against vaccine-preventable diseases. Contradictory to this, routine vaccine appointments were delayed or canceled, as the general acceptance of vaccination among parents fell. Moreover, the availability of certain vaccines was also limited^40–43^. Hypothetically, these developments may have contributed to an increased susceptibility to vaccine-preventable infections. However, we do not have vaccination rates for the patients included in the study. Intracranial complications of sinonasal and otogenic infections can also be supported by a delay in adequate therapy^44^. During the pandemic, visits to pediatricians were avoided whenever possible to protect both children and healthcare staff. Sick notes, prescriptions, and even medical advice were frequently provided by telephone or without an in-person consultation. Consequently, infections requiring treatment may have gone unrecognized. Regarding the duration of school closures in the participating countries, we observed no clear association between the increase in the respective case frequencies per semiannual period and the duration of school closures. Further investigations into this relationship are warranted.

The main strength of this multicenter study is the large number of patients included across several international centers, constituting, to our knowledge, the largest international cohort focusing on intracranial complications of sinogenic and otogenic origin in the context of the COVID-19 pandemic. This broad, cross-country recruitment allows tentative conclusions regarding epidemiologic developments. However, the observed post-pandemic increase in case numbers cannot be attributed solely to pandemic-related factors, as our data already show a slight increase in cases per half-year between 2015 and 2019, which cannot be fully explained within the scope of this study.

Further limitations arise from the retrospective design and the dependence on the quality and consistency of documentation in the participating centers. Incomplete datasets, particularly regarding virological findings, precluded meaningful analyses of viral infections, and the limited availability of laboratory parameters restricted a more detailed assessment of the patients’ immune response. Another methodological challenge concerns the epidemiological data. A true incidence rate requires an appropriate population denominator (e.g., the total number of pediatric hospitalizations or the total number of cases of uncomplicated sinusitis or otitis media during the respective study periods). As these data were unfortunately unavailable, we used the terms “cases per semiannual” and “case frequency” throughout the study.

## Conclusion

Our study further supports the observation of a highly significant increase in cases of intracranial complications of sinogenic and otogenic origin as well as intraorbital complications of sinusitis in the context of covid pandemic. The sharp increase in frequencies was not accompanied by changes in patient characteristics and a direct relation to a SARS-Cov-2 infection could not be established based on our data, though such an association could not be ruled out either. Pandemic restrictions might have had impacts on the epidemiology and burden of pediatric respiratory infections potentially leading to intracranial complications. This might have occurred as a result of NPI-induced lack of immune stimulation. Furthermore, disruptions to routine health services and reductions in vaccination programs may have contributed to the development described. The study presented here should highlight the importance of global respiratory infection research and infection surveillance programs, as well as open data sharing, maintaining vaccine development, and use in the pediatric population.

## Supplementary Information

Below is the link to the electronic supplementary material.

Supplementary Material 1

## Data Availability

The anonymized raw data sets generated from the individual participating study centers in European and non-European countries that were analyzed during the current study are available from the corresponding author on reasonable request.

## Abbreviations

BMI: Body mass index
CSVT: Cerebral sinus vein thrombosis
EIN: Emergency Infections Network
EPOS: European Position Paper on Rhinosinusitis and Nasal Polyps
ESPN: European Society of Pediatric Neurosurgery
ICSO: Intracranial complications of sinusitis and otitis
NPIs: Non-pharmacological interventions
PCR: Polymerase chain reaction
RSV: Respiratory Syncytial Virus
