# Supplementary material for: A multicenter retrospective study on severe complications of acute otitis media and sinusitis in healthy children in the context of covid pandemic – CAOS-kids
Source: Sci Rep. 2026 Aug 21;16:26343. doi: 10.1038/s41598-026-67005-y (PMC13498597; doi:10.1038/s41598-026-67005-y)
Supplement: Supplementary file 1 [file 41598_2026_67005_MOESM1_ESM.pdf]

A multicenter retrospective study on severe complications of acute otitis media and sinusitis in healthy children in the context of covid pandemic – CAOS-kids

Kornelia E. Andorfer, Torstein Grønseth, Juned Zafar Butt, Randall A. Bly, Jeremy S. Ruthberg , Michael Habenbacher, Peter Valentin Tomazic, Fatemeh Kashani, Maria Teresa Arcuri, Bresciani Lorenzo, Marie Gisselsson-Solén , Isabella Sjölander, Sara Zenk, Valentinos Sofokleous, Ivan Delchev, Florian Zeman, Christopher Bohr, Kathrin Renner

Leukocyte composition

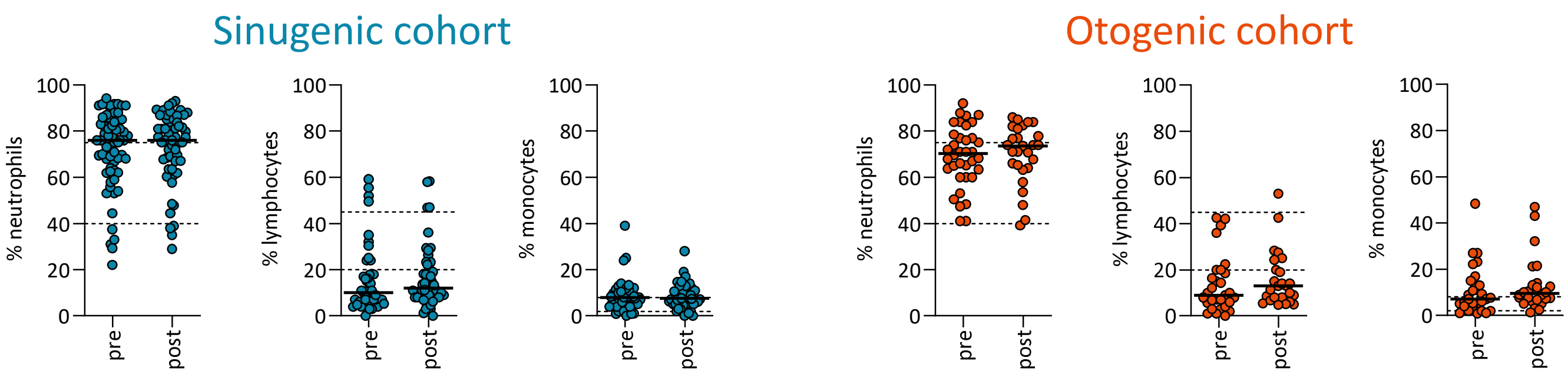

Supplementary Fig. 1. Peripheral blood leukocyte composition in children diagnosed with intracranial sinogenic or otogenic complications. The distribution of the leukocyte populations in the peripheral blood was determined in a routine differential blood count. Displayed are single data points and median values, dashed lines show age matched reference range for healthy children. Statistical significance was tested using the Mann Whitney test.

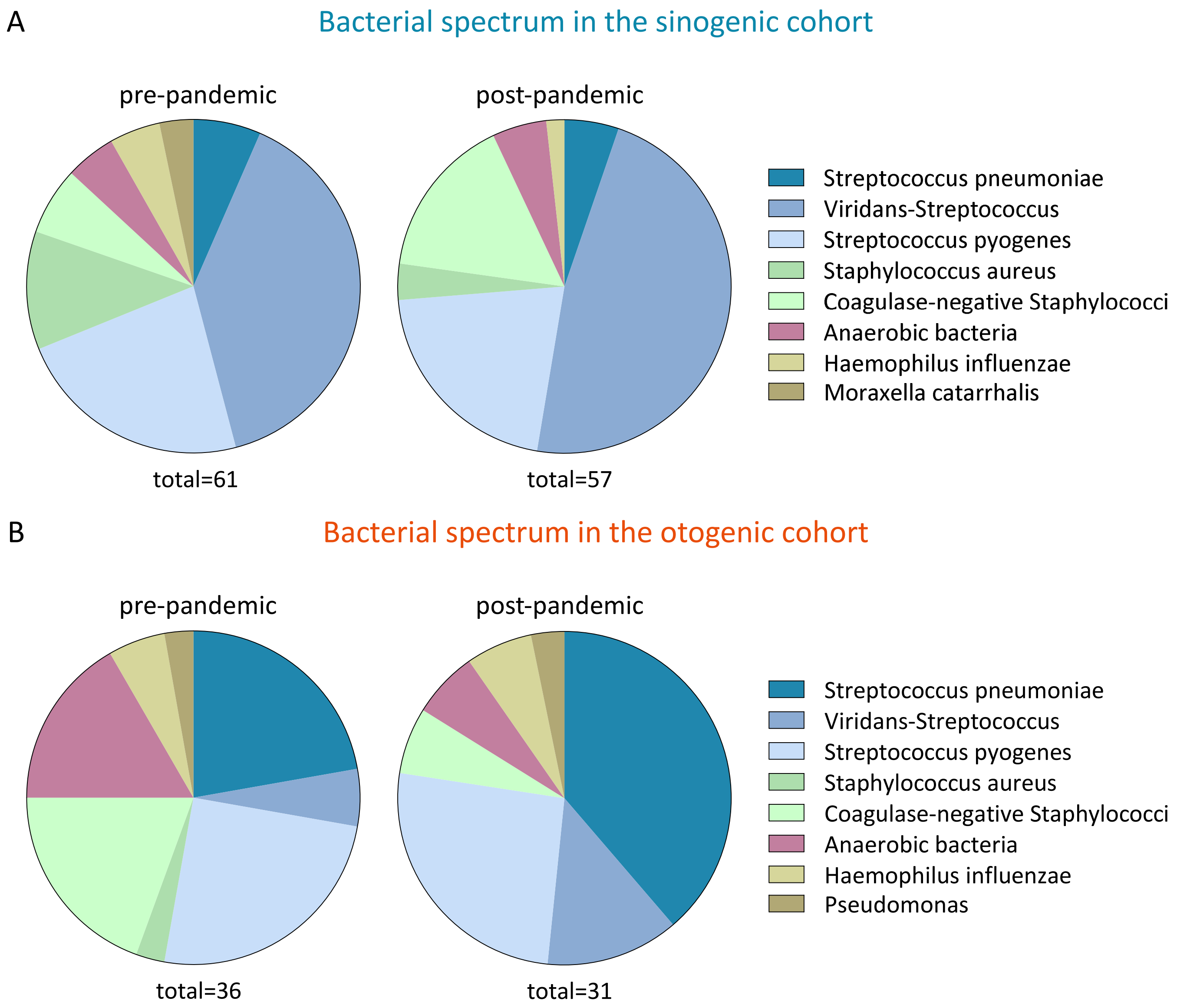

Supplementary Fig. 2. Type and frequency of bacterial strains and species did not differ in the pre-pandemic and post-pandemic subgroups. Data for (**a**) sinogenic and (**b**) the otogenic pre- and post-pandemic subgroups are displayed.

sinogenic cohort					
demographic data	total (%)	pre-pandemic (%)	pandemic (%)	post-pandemic (%)	p-value pre vs. post
number of cases	34	14 (41)	7 (20)	13 (38)	
gender					0.44
female	14 (41)	4 (29)	4 (57)	6 (46)	
male	20 (59)	10 (71)	3 (43)	7 (54)	
age (IQR)		8.5 (4.8-10.0)	6.0 (2.0-13.0)	10 (6.0-11.5)	0.27

Supplementary Table 1. Demographics of the second Swedish cohort comprising cases from 2017 through 2023. For age median is displayed and inter quartile range (IQR).

sinogenic cohort						
country	pre-pandemic cases	pre-pandemic frequency	post-pandemic cases	post-pandemic frequency	Delta frequencies	weeks partial/full school closure
Germany	17	1.06	21	7.00	5.94	38
USA	20	1.25	16	5.33	4.08	71
Norway	30	1.88	16	5.33	3.45	29
Sweden	19	2.67	17	5.67	3.00	24
Greece	0	0	4	1.33	1.33	37
Austria	11	0.69	8	2.67	1.98	39
Italy	3	0.19	3	1.00	0.81	38

Supplementary Table 2. Calculation of frequencies on a per-country base and weeks of school closure. For Sweden both cohorts were combined.
